# Supplementary material for: Differences in the relationships between interoceptive sensibility and self-objectification in women with high and low body dissatisfaction: A network analysis
Source: PLoS One. 2025 May 28;20(5):e0323524. doi: 10.1371/journal.pone.0323524 (PMC12118901; doi:10.1371/journal.pone.0323524)

**S1 Fig.** Edge weight accuracy plot for high and low body dissatisfaction group.


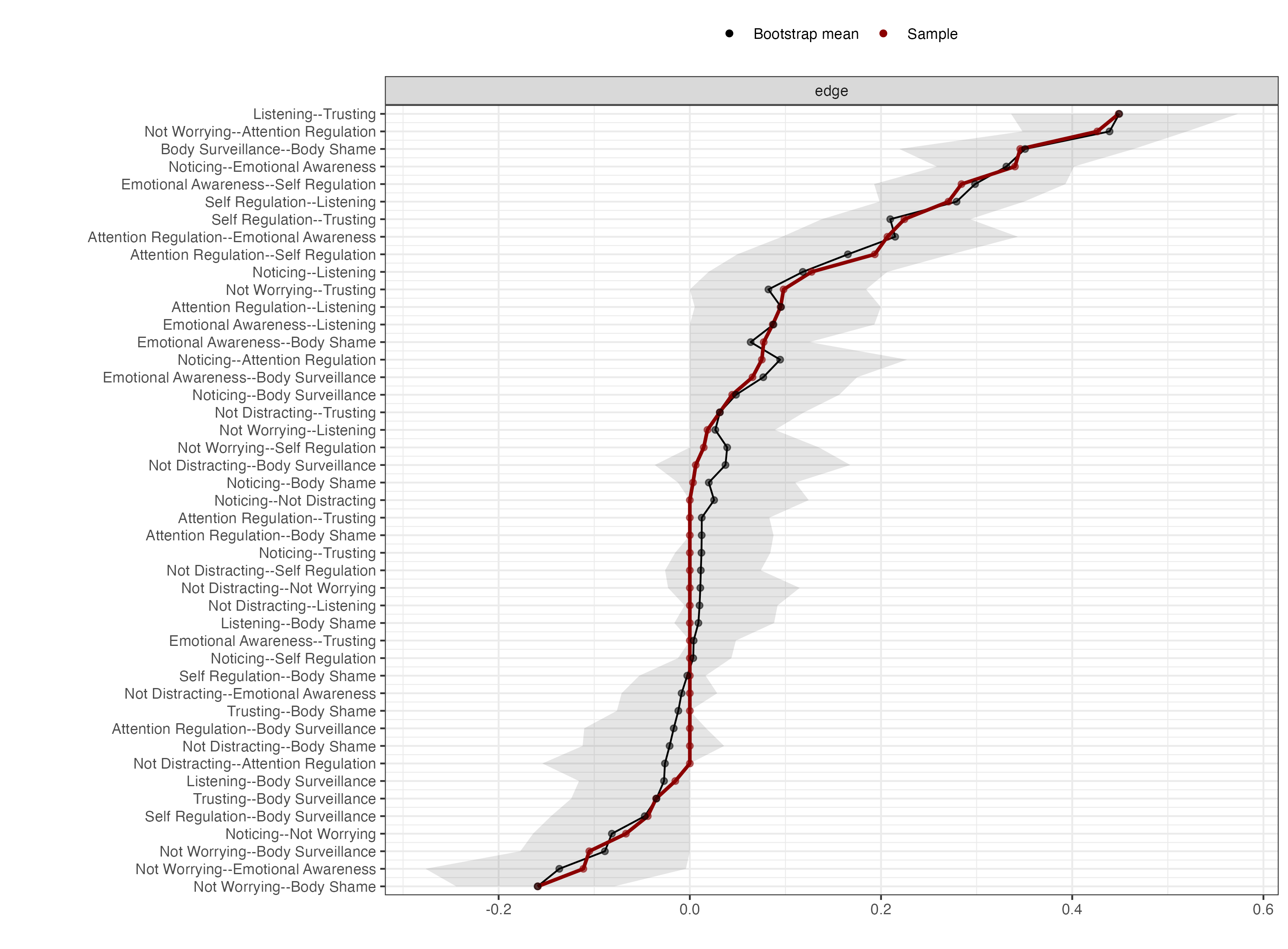
High body dissatisfaction Low body dissatisfaction


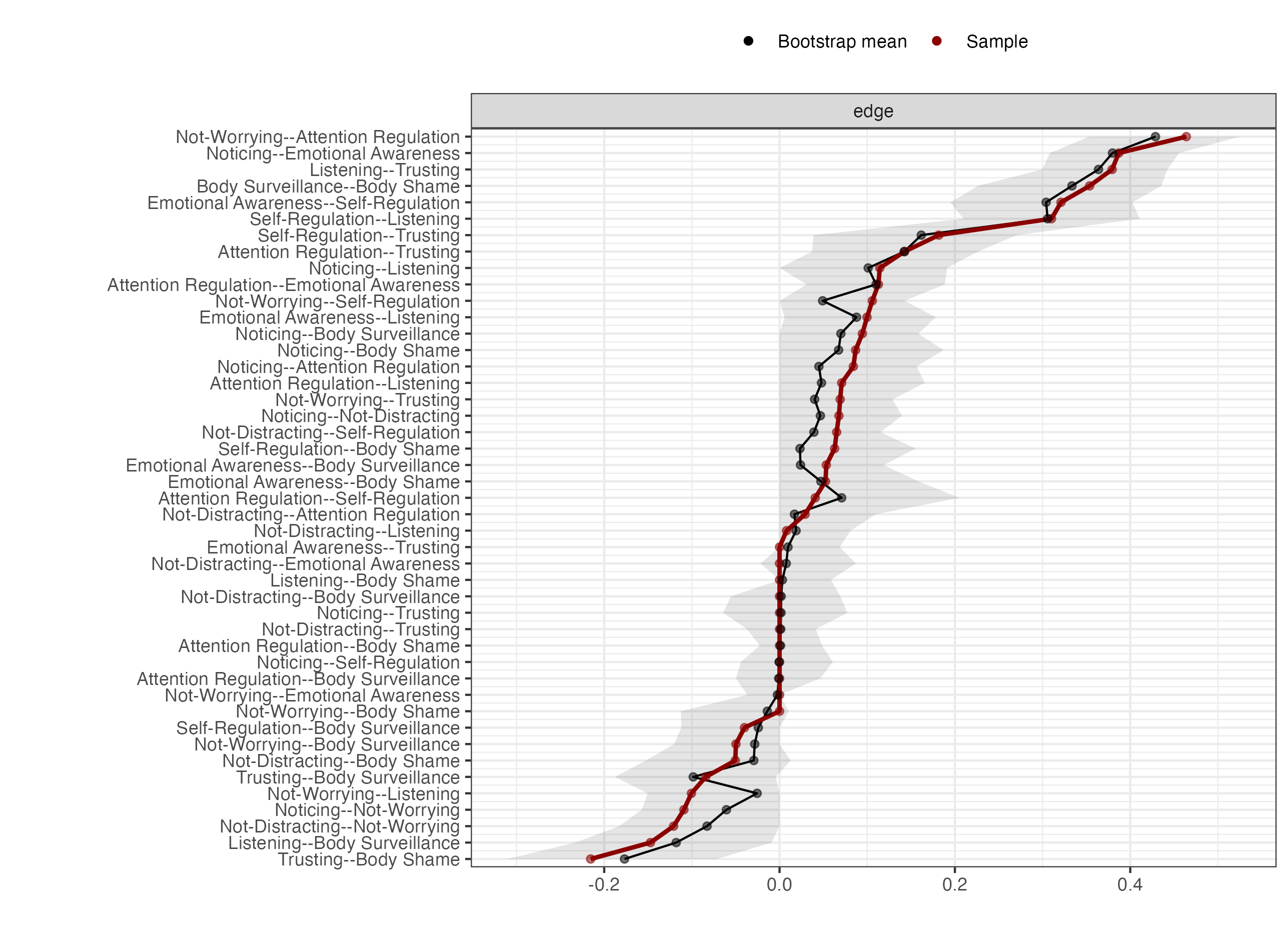

Supplement: S1 Fig — (DOCX) [file pone.0323524.s001.docx]
