# Supplementary material for: Differences in the relationships between interoceptive sensibility and self-objectification in women with high and low body dissatisfaction: A network analysis
Source: PLoS One. 2025 May 28;20(5):e0323524. doi: 10.1371/journal.pone.0323524 (PMC12118901; doi:10.1371/journal.pone.0323524)
Supplement: S1 Table — (DOCX) [file pone.0323524.s002.docx]

**S1 Table**. Demographic information for all participants (N=1372) in the study

| **Country** | **Number of participants** |
| --- | --- |
| Albania | 1 |
| Argentina | 1 |
| Australia | 17 |
| Austria | 3 |
| Belgium | 7 |
| Brazil | 4 |
| Bulgaria | 1 |
| Canada | 30 |
| Chile | 3 |
| China | 2 |
| Costa Rica | 1 |
| Cuba | 1 |
| Cyprus | 1 |
| Czech Republic | 7 |
| El Salvador | 1 |
| Estonia | 8 |
| Eswatini | 1 |
| Ethiopia | 1 |
| Finland | 6 |
| France | 11 |
| Germany | 18 |
| Greece | 32 |
| Honduras | 1 |
| Hong Kong | 3 |
| Hungary | 18 |
| Iceland | 1 |
| India | 1 |
| Iran | 1 |
| Ireland | 11 |
| Israel | 4 |
| Italy | 58 |
| Japan | 1 |
| Kenya | 1 |
| Latvia | 3 |
| Lebanon | 2 |
| Lithuania | 1 |
| Luxembourg | 1 |
| Malaysia | 3 |
| Mexico | 68 |
| Netherlands | 9 |
| Nigeria | 6 |
| Norway | 1 |
| Pakistan | 1 |
| Poland | 96 |
| Portugal | 114 |
| Romania | 1 |
| Russia | 4 |
| Saudi Arabia | 1 |
| Slovenia | 7 |
| South Africa | 222 |
| South Korea | 2 |
| Spain | 31 |
| Sri Lanka | 1 |
| Sweden | 2 |
| Switzerland | 2 |
| Turkey | 7 |
| UK | 443 |
| USA | 66 |
| Ukraine | 2 |
| Uzbekistan | 1 |
| Venezuela | 1 |
| Zambia | 1 |
| Zimbabwe | 4 |
| Indonesia | 2 |
