# Supplementary material for: Differences in the relationships between interoceptive sensibility and self-objectification in women with high and low body dissatisfaction: A network analysis
Source: PLoS One. 2025 May 28;20(5):e0323524. doi: 10.1371/journal.pone.0323524 (PMC12118901; doi:10.1371/journal.pone.0323524)
Supplement: S2 Table — (DOCX) [file pone.0323524.s003.docx]

| **S2 Table.**  Spearman-product correlation matrix for raw dataset. | | | | | | | | | | | | | | | | | | | | | | | |
| --- | --- | --- | --- | --- | --- | --- | --- | --- | --- | --- | --- | --- | --- | --- | --- | --- | --- | --- | --- | --- | --- | --- | --- |
|  | |  | | **Self-surveillance** | | **Body Shame** | | **Noticing** | | **Not Distracting** | | **Not Worrying** | | **Attention Regulation** | | **Emotional Awareness** | | **Self Regulatio n** | | **Listening** | | **Trusting** | |
| Self-surveillance |  | Pearson's r |  | — |  |  |  |  |  |  |  |  |  |  |  |  |  |  |  |  |  |  |  |
|  |  | p-value |  | — |  |  |  |  |  |  |  |  |  |  |  |  |  |  |  |  |  |  |  |
| Body Shame |  | Pearson's r |  | 0.457 | *** | — |  |  |  |  |  |  |  |  |  |  |  |  |  |  |  |  |  |
|  |  | p-value |  | < .001 |  | — |  |  |  |  |  |  |  |  |  |  |  |  |  |  |  |  |  |
| Noticing |  | Pearson's r |  | 0.113 | * | 0.151 | ** | — |  |  |  |  |  |  |  |  |  |  |  |  |  |  |  |
|  |  | p-value |  | 0.035 |  | 0.005 |  | — |  |  |  |  |  |  |  |  |  |  |  |  |  |  |  |
| Not Distracting |  | Pearson's r |  | -0.026 |  | -0.070 |  | 0.136 | * | — |  |  |  |  |  |  |  |  |  |  |  |  |  |
|  |  | p-value |  | 0.636 |  | 0.197 |  | 0.011 |  | — |  |  |  |  |  |  |  |  |  |  |  |  |  |
| Not Worrying |  | Pearson's r |  | -0.158 | ** | -0.106 | * | -0.036 |  | -0.121 | * | — |  |  |  |  |  |  |  |  |  |  |  |
|  |  | p-value |  | 0.003 |  | 0.048 |  | 0.501 |  | 0.024 |  | — |  |  |  |  |  |  |  |  |  |  |  |
| Attention Regulation |  | Pearson's r |  | -0.130 | * | -0.083 |  | 0.225 | *** | 0.045 |  | 0.533 | *** | — |  |  |  |  |  |  |  |  |  |
|  |  | p-value |  | 0.015 |  | 0.125 |  | < .001 |  | 0.407 |  | < .001 |  | — |  |  |  |  |  |  |  |  |  |
| Emotional awareness |  | Pearson's r |  | 0.018 |  | 0.088 |  | 0.535 | *** | 0.096 |  | 0.112 | * | 0.348 | *** | — |  |  |  |  |  |  |  |
|  |  | p-value |  | 0.732 |  | 0.104 |  | < .001 |  | 0.075 |  | 0.037 |  | < .001 |  | — |  |  |  |  |  |  |  |
| Self regulation |  | Pearson's r |  | -0.200 | *** | -0.037 |  | 0.280 | *** | 0.127 | * | 0.259 | *** | 0.384 | *** | 0.553 | *** | — |  |  |  |  |  |
|  |  | p-value |  | < .001 |  | 0.495 |  | < .001 |  | 0.018 |  | < .001 |  | < .001 |  | < .001 |  | — |  |  |  |  |  |
| Listening |  | Pearson's r |  | -0.302 | *** | -0.144 | ** | 0.324 | *** | 0.113 | * | 0.112 | * | 0.359 | *** | 0.447 | *** | 0.611 | *** | — |  |  |  |
|  |  | p-value |  | < .001 |  | 0.007 |  | < .001 |  | 0.035 |  | 0.037 |  | < .001 |  | < .001 |  | < .001 |  | — |  |  |  |
| Trusting |  | Pearson's r |  | -0.340 | *** | -0.325 | *** | 0.167 | ** | 0.048 |  | 0.272 | *** | 0.408 | *** | 0.322 | *** | 0.524 | *** | 0.627 | *** | — |  |
|  |  | p-value |  | < .001 |  | < .001 |  | 0.002 |  | 0.373 |  | < .001 |  | < .001 |  | < .001 |  | < .001 |  | < .001 |  | — |  |
| Note. * p < .05, ** p < .01, *** p < .001 | | | | | | | | | | | | | | | | | | | | | | | |
|  | | | | | | | | | | | | | | | | | | | | | | | |
