# Supplementary material for: Differences in the relationships between interoceptive sensibility and self-objectification in women with high and low body dissatisfaction: A network analysis
Source: PLoS One. 2025 May 28;20(5):e0323524. doi: 10.1371/journal.pone.0323524 (PMC12118901; doi:10.1371/journal.pone.0323524)
Supplement: S3 Table — (DOCX) [file pone.0323524.s004.docx]

| S3 Table. Spearman-product correlation matrix on age regressed dataset. To control for the effect of age, each variable was regressed by age and the obtained standardized residuals were correlated | | | | | | | | | | | | | | | | | | | | | | | |
| --- | --- | --- | --- | --- | --- | --- | --- | --- | --- | --- | --- | --- | --- | --- | --- | --- | --- | --- | --- | --- | --- | --- | --- |
|  | |  | | **Self-surveillance** | | **Body Shame** | | **Noticing** | | **Not Distracting** | | **Not Worrying** | | **Attention Regulation** | | **Emotional Awareness** | | **Self Regulatio n** | | **Listening** | | **Trusting** | |
| Self-surveillance |  | Pearson's r |  | — |  |  |  |  |  |  |  |  |  |  |  |  |  |  |  |  |  |  |  |
|  |  | p-value |  | — |  |  |  |  |  |  |  |  |  |  |  |  |  |  |  |  |  |  |  |
| Body Shame |  | Pearson's r |  | 0.436 | *** | — |  |  |  |  |  |  |  |  |  |  |  |  |  |  |  |  |  |
|  |  | p-value |  | < .001 |  | — |  |  |  |  |  |  |  |  |  |  |  |  |  |  |  |  |  |
| Noticing |  | Pearson's r |  | 0.093 | * | 0.069 |  | — |  |  |  |  |  |  |  |  |  |  |  |  |  |  |  |
|  |  | p-value |  | 0.014 |  | 0.068 |  | — |  |  |  |  |  |  |  |  |  |  |  |  |  |  |  |
| Not Distracting |  | Pearson's r |  | -0.049 |  | -0.030 |  | 0.061 |  | — |  |  |  |  |  |  |  |  |  |  |  |  |  |
|  |  | p-value |  | 0.195 |  | 0.433 |  | 0.104 |  | — |  |  |  |  |  |  |  |  |  |  |  |  |  |
| Not Worrying |  | Pearson's r |  | -0.207 | *** | -0.168 | *** | 0.021 |  | -0.026 |  | — |  |  |  |  |  |  |  |  |  |  |  |
|  |  | p-value |  | < .001 |  | < .001 |  | 0.586 |  | 0.487 |  | — |  |  |  |  |  |  |  |  |  |  |  |
| Attention Regulation |  | Pearson's r |  | -0.094 | * | -0.120 | ** | 0.304 | *** | -0.002 |  | 0.545 | *** | — |  |  |  |  |  |  |  |  |  |
|  |  | p-value |  | 0.013 |  | 0.002 |  | < .001 |  | 0.949 |  | < .001 |  | — |  |  |  |  |  |  |  |  |  |
| Emotional awareness |  | Pearson's r |  | 0.057 |  | 0.061 |  | 0.516 | *** | 0.019 |  | 0.112 | ** | 0.431 | *** | — |  |  |  |  |  |  |  |
|  |  | p-value |  | 0.129 |  | 0.108 |  | < .001 |  | 0.607 |  | 0.003 |  | < .001 |  | — |  |  |  |  |  |  |  |
| Self regulation |  | Pearson's r |  | -0.135 | *** | -0.099 | ** | 0.332 | *** | 0.086 | * | 0.296 | *** | 0.479 | *** | 0.581 | *** | — |  |  |  |  |  |
|  |  | p-value |  | < .001 |  | 0.009 |  | < .001 |  | 0.022 |  | < .001 |  | < .001 |  | < .001 |  | — |  |  |  |  |  |
| Listening |  | Pearson's r |  | -0.187 | *** | -0.142 | *** | 0.385 | *** | 0.084 | * | 0.216 | *** | 0.434 | *** | 0.484 | *** | 0.642 | *** | — |  |  |  |
|  |  | p-value |  | < .001 |  | < .001 |  | < .001 |  | 0.027 |  | < .001 |  | < .001 |  | < .001 |  | < .001 |  | — |  |  |  |
| Trusting |  | Pearson's r |  | -0.230 | *** | -0.233 | *** | 0.249 | *** | 0.074 | * | 0.303 | *** | 0.420 | *** | 0.367 | *** | 0.573 | *** | 0.672 | *** | — |  |
|  |  | p-value |  | < .001 |  | < .001 |  | < .001 |  | 0.050 |  | < .001 |  | < .001 |  | < .001 |  | < .001 |  | < .001 |  | — |  |
| Note. * p < .05, ** p < .01, *** p < .001 | | | | | | | | | | | | | | | | | | | | | | | |
|  | | | | | | | | | | | | | | | | | | | | | | | |
