# Supplementary material for: Differences in the relationships between interoceptive sensibility and self-objectification in women with high and low body dissatisfaction: A network analysis
Source: PLoS One. 2025 May 28;20(5):e0323524. doi: 10.1371/journal.pone.0323524 (PMC12118901; doi:10.1371/journal.pone.0323524)
Supplement: Attachment — Submitted filename: Response to reviewers.docx [file pone.0323524.s006.docx]

**Response to Reviewers**

Dear Reviewers,

Thank you for your thoughtful comments, which I have addressed below point-by-point. I have identified the location of each change in the manuscript in red and copied and pasted the revised text under the respective comments. I have responded individually to each reviewer’s comments, separating responses for Reviewer 1 and Reviewer 2. I believe these recommendations have enhanced the manuscript, and we hope you find the revision acceptable for publication.

**Reviewer #1:** It is an interesting article which reaches interesting conclusions.

The limitations of the study that I observed (for example, the possible differences in the results considering age groups or the different aspects of interoception) are pointed out in the limitations of this study, therefore, they are rectified. However, it would have been interesting to point out also in the limitations the possible cultural differences that could influence interoception and body dissatisfaction, since it is known that there is a cultural influence on this and the sample is composed of women from different countries. Also to report in a table the demographic data of the participants considering the above mentioned.

These findings open the door to new studies on the subject and the clinical management of body dissatisfaction.

**Response**

Thank you for your positive feedback and helpful suggestions.

In response to your comment:

- I have now updated the limitations section to explicitly acknowledge that cultural differences may influence interoceptive sensibility and body dissatisfaction. I have also highlighted that research still finds an antagonistic relationship between them cross-culturally. Additionally, a demographic table with all the ethnicities of the participants is located in Supplementary Table 1.

‘Given the diverse nature of the sample, cultural differences in body dissatisfaction and interoceptive sensibility may have influenced the findings. For instance, individuals from non-Western cultures tend to report higher interoceptive sensibility [74] and may express body dissatisfaction in culturally specific ways, such as concerns with facial features [75] or skin tone [76]. Nonetheless, cross-cultural research suggests that the antagonistic relationship between body image and interoception is observed across diverse ethnic groups [37, 77].’

**Reviewer #2**: Thank you for the opportunity to review the article entitled "Differences in the Relationships Between Interoceptive Sensibility and Self-Objectification in Women with High and Low Body Dissatisfaction: A Network Analysis". The work is well written and organized, focusing on a central theme currently. I believe the work is of high quality. I will only highlight two aspects regarding limitations and future directions of research. The sampling through a rewarded platform, which uses only self-report questionnaires (especially for self-report of absence of eating disorders and for interoceptive abilities) is a limitation. Interoception is a complex construct and, although a widely used and validated instrument was used, it may not be best captured in a self-report. Furthermore, the age range of the sample is very wide. Young adults may have different social pressures than older adults, and there may be different nuances. It would be interesting to investigate these aspects in the future also in a younger sample: adolescents and emerging adulthood. Overall I think the work is very interesting and, after adding these limitations, it can be an important contribution to the literature.

**Response**

Thank you for your positive feedback and for drawing attention to these important limitations.

We agree that interoception is a multidimensional construct, and we have addressed this in the “Conclusions and Limitations” section of the manuscript, as highlighted below:

Interoception is a multidimensional construct comprising interoceptive accuracy, interoceptive sensibility, and interoceptive awareness, according to the tripartite model of interoception [70]. However, these different constructs are not proxies for one another, and numerous studies have demonstrated independence between subjective self-report measures of interoception (i.e interoceptive sensibility) and objective measurements of interoception (e.g. interoceptive accuracy) [71–73]. Therefore, future research should investigate relationships between self-objectification and other interoception dimensions to better understand these relationships within the context of body dissatisfaction.

We also agree that the age range of the sample is wide, and there are age-related variations in body dissatisfaction. As such, we have now included the following sentence to the “Conclusions and Limitations” section:

Additionally, the wide age range of the sample may have introduced age-related effects, as younger individuals often report greater appearance-based body dissatisfaction due to societal pressures [78], while older individuals may experience more dissatisfaction with their body functionality as opposed to appearance [79].

To highlight the point you made about the need to investigate the relationships between interoceptive sensibility and body dissatisfaction in younger individuals, I have also added this statement to the “Conclusions and Limitations” section:

Given the complex nature of body image and interoceptive sensibility, future longitudinal studies should consider using network analysis to examine their dynamic relationships throughout the lifespan. This is particularly important during adolescence and emerging adulthood, which are critical periods for the development of eating disorders.
